# Supplementary material for: Tensor network simulation of multi-environmental open quantum dynamics via machine learning and entanglement renormalisation
Source: Nat Commun. 2019 Mar 5;10:1062. doi: 10.1038/s41467-019-09039-7 (PMC6401190; doi:10.1038/s41467-019-09039-7)
Supplement: Supplementary file 1 — Supplementary Information [file 41467_2019_9039_MOESM1_ESM.pdf]

Supplementary information for:
Tensor Network Simulation of Multi-Environmental Open Quantum Dynamics via Machine Learning and Entanglement Renormalisation

Florian A. Y. N. Schröder *et al.*

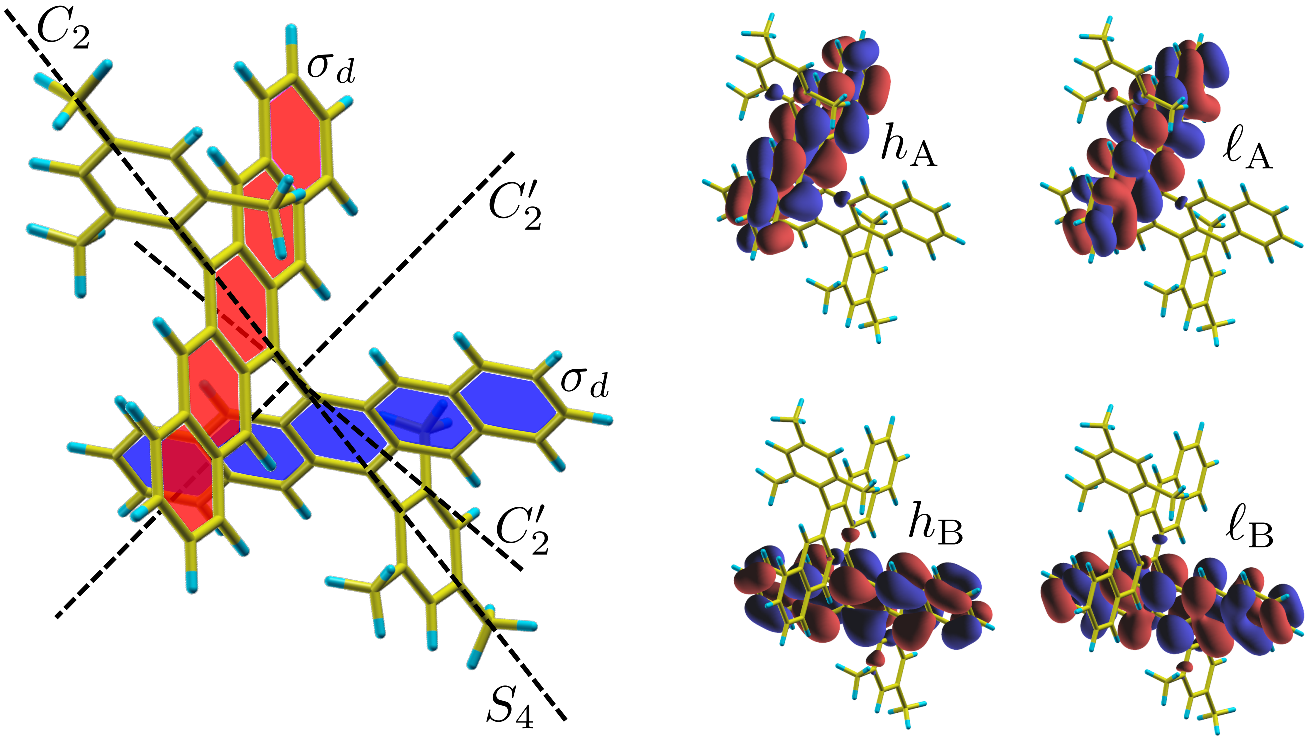

Supplementary Figure 1. (a) DP-Mes structure with symmetry elements of the D_{2d} point group, and (b) frontier molecular orbitals used in calculations and this supplemental note. Note that extremal hydrogens of mesitylene side-groups in (a) weakly break the point group symmetry. However, this is irrelevant for the low-energy excitations since the frontier orbitals shown in (b) firmly reside on the pentacene units.

Supplementary Note 1 - Electronic structure calculations

Off-diagonal couplings and symmetries. To first order in nuclear displacements, the matrix elements of the diabatic potential matrix between electronic states i and j are given by[1]

$$W_{n,(ij)} = \left\langle i \left| \frac{\partial \hat{H}_{el}}{\partial Q_n} \right| j \right\rangle , \tag{1}$$

where the Q_n represent normal-mode coordinates of the ground state. The diagonal elements are simply the adiabatic forces (expressed in terms of normal coordinates), which we obtain directly from DFT.

For the purposes of the symmetry analysis and evaluation of the off-diagonal coupling vectors we employ an approximate multi-Supplementary Reference description of the electronic states, following Supplementary Reference 2. This uses a small active space comprised of the four orbitals h_{A}, h_{B}, ℓ_{A}, ℓ_{B}, namely the monomer-localised HOMOs/LUMOs corresponding to the two pentacene units that make up DP-Mes (see Supplementary Figure 1). As can be seen from the figure this set of localised orbitals is already orthogonal due to symmetry, meaning that a separate Löwdin orthogonalisation step is not necessary. In this set the ground state can be represented as the single determinant $|h_{\mathrm{A}}\alpha h_{\mathrm{A}}\beta h_{\mathrm{B}}\alpha h_{\mathrm{B}}\beta|$, where α and β label the electron spins. Furthermore, we have the locally excited states of each pentacene unit:

$$\mathrm{LE_A} = \frac{1}{\sqrt{2}} \left[|h_{\mathrm{A}}\alpha \ell_{\mathrm{A}}\beta h_{\mathrm{B}}\alpha h_{\mathrm{B}}\beta| - |h_{\mathrm{A}}\beta \ell_{\mathrm{A}}\alpha h_{\mathrm{B}}\alpha h_{\mathrm{B}}\beta| \right] , \tag{2}$$

$$\mathrm{LE_B} = \frac{1}{\sqrt{2}} \left[|h_{\mathrm{A}}\alpha h_{\mathrm{A}}\beta h_{\mathrm{B}}\alpha \ell_{\mathrm{B}}\beta| - |h_{\mathrm{A}}\alpha h_{\mathrm{A}}\beta h_{\mathrm{B}}\beta \ell_{\mathrm{B}}\alpha| \right] , \tag{3}$$

Supplementary Table I. Excitation energies and symmetry classification of electronic states.

state	E (eV)	irrep	E	$2S_4$	C_2	$2C_2'$	$2\sigma_d$
GS	0.00	A_1	+1	+1	+1	+1	+1
TT	1.83	A_1	+1	+1	+1	+1	+1
LE$^+$	2.07	B_2	+1	−1	+1	−1	+1
LE$^-$	2.20	A_1	+1	+1	+1	+1	+1
CT$^+$	2.75	A_2	+1	+1	+1	−1	−1
CT$^-$	2.76	B_1	+1	−1	+1	+1	−1

Supplementary Table II. Product table of 1D-irreps of D_{2d}.

	A_1	A_2	B_1	B_2
A_1	A_1	A_2	B_1	B_2
A_2	A_2	A_1	B_2	B_1
B_1	B_1	B_2	A_1	A_2
B_2	B_2	B_1	A_2	A_1

as well as the corresponding CT states where the excited electron is transferred to the other pentacene unit, respectively:

$$\mathrm{CT_A} = \frac{1}{\sqrt{2}}\left[|h_\mathrm{A}\alpha\ell_\mathrm{B}\beta h_\mathrm{B}\alpha h_\mathrm{B}\beta| - |h_\mathrm{A}\beta\ell_\mathrm{B}\alpha h_\mathrm{B}\alpha h_\mathrm{B}\beta|\right], \tag{4}$$

$$\mathrm{CT_B} = \frac{1}{\sqrt{2}}\left[|h_\mathrm{A}\alpha h_\mathrm{A}\beta h_\mathrm{B}\alpha\ell_\mathrm{A}\beta| - |h_\mathrm{A}\alpha h_\mathrm{A}\beta h_\mathrm{B}\beta\ell_\mathrm{A}\alpha|\right]. \tag{5}$$

To obtain states that transform as irreducible representations of the point group we need to perform an (anti)symmetrisation step:

$$\mathrm{LE}^{\pm} = \frac{1}{\sqrt{2}}\left[\mathrm{LE_A} \pm \mathrm{LE_B}\right], \tag{6}$$

$$\mathrm{CT}^{\pm} = \frac{1}{\sqrt{2}}\left[\mathrm{CT_A} \pm \mathrm{CT_B}\right]. \tag{7}$$

Finally, the correlated triplet pair TT can be expressed as:

$$\begin{aligned}\mathrm{TT} = \frac{1}{\sqrt{3}}[&|h_\mathrm{A}\alpha\ell_\mathrm{A}\alpha h_\mathrm{B}\beta\ell_\mathrm{B}\beta| + |h_\mathrm{A}\beta\ell_\mathrm{A}\beta h_\mathrm{B}\alpha\ell_\mathrm{B}\alpha| \\ &-\frac{1}{2}(|h_\mathrm{A}\alpha\ell_\mathrm{A}\beta h_\mathrm{B}\alpha\ell_\mathrm{B}\beta| + |h_\mathrm{A}\alpha\ell_\mathrm{A}\beta h_\mathrm{B}\beta\ell_\mathrm{B}\alpha| \\ &+|h_\mathrm{A}\beta\ell_\mathrm{A}\alpha h_\mathrm{B}\alpha\ell_\mathrm{B}\beta| + |h_\mathrm{A}\beta\ell_\mathrm{A}\alpha h_\mathrm{B}\beta\ell_\mathrm{B}\alpha|)].\end{aligned} \tag{8}$$

Using this picture one can now classify the electronic states according to irreducible representations of the point group. First, one considers how the minimal orbital basis h_A, h_B, ℓ_A, ℓ_B transforms under the symmetry operations of D_{2d}. For example a C_2 rotation about the principle axis induces the mapping $h_\mathrm{A} \to -h_\mathrm{A}$, $h_\mathrm{B} \to -h_\mathrm{B}$, $\ell_\mathrm{A} \to -\ell_\mathrm{A}$ and $\ell_\mathrm{B} \to -\ell_\mathrm{B}$. By considering these transformations under all symmetry operations and substituting into the minimal-basis wave functions of the electronic states one obtains the right hand part of Supplementary Table I. The sign changes of the wave functions under the symmetry operations then directly determine the respective symmetry label.

To evaluate the diabatic potential matrix W to first order we can now go ahead and substitute the frontier orbital representations of the electronic states. Application of the Slater-Condon rules and some algebra then yields the

following off-diagonal coupling contributions:

$$W_{(\mathrm{LE^+LE^-})} = \frac{1}{2}\left[W_{(h_\mathrm{B}h_\mathrm{B})} - W_{(h_\mathrm{A}h_\mathrm{A})} - W_{(\ell_\mathrm{B}\ell_\mathrm{B})} + W_{(\ell_\mathrm{A}\ell_\mathrm{A})}\right] \tag{9}$$

$$W_{(\mathrm{CT^+CT^-})} = \frac{1}{2}\left[W_{(h_\mathrm{B}h_\mathrm{B})} - W_{(h_\mathrm{A}h_\mathrm{A})} + W_{(\ell_\mathrm{B}\ell_\mathrm{B})} - W_{(\ell_\mathrm{A}\ell_\mathrm{A})}\right] \tag{10}$$

$$W_{(\mathrm{LE^+CT^+})} = W_{(\ell_\mathrm{A}\ell_\mathrm{B})} - W_{(h_\mathrm{A}h_\mathrm{B})} \tag{11}$$

$$W_{(\mathrm{LE^-CT^-})} = W_{(\ell_\mathrm{A}\ell_\mathrm{B})} + W_{(h_\mathrm{A}h_\mathrm{B})} \tag{12}$$

$$W_{(\mathrm{TT\ CT^\pm})} = \frac{\sqrt{3}}{2}\left[W_{(\ell_\mathrm{A}h_\mathrm{B})} \pm W_{(h_\mathrm{A}\ell_\mathrm{B})}\right] \tag{13}$$

The matrix elements $W_{(ab)}$ between single orbitals a, b are now simply the Coulombic forces on the nuclei due to the overlap density $a(\mathbf{r})b(\mathbf{r})$. It should be pointed out that the first-order coupling vectors cannot have contributions from two-electron matrix elements since the electron-electron Coulomb interaction is independent of the nuclear coordinates and vanishes when taking the derivative in Supplementary equation (1). Any vibrational mode can only give a non-zero contribution to the coupling between two electronic states if the product of the respective irreducible representations yields the totally symmetric representation A_1. For example modes contributing to the coupling between LE$^+$ (B_2) and CT$^+$ (A_2) necessarily transform as B_1 (see Supplementary Figure 2 and Supplementary Table II).

Limitations. There are a number of limitations of the methods and approximations described. These include treating the normal modes in the harmonic regime, and only accounting for the coupling between vibrations and electronic states to first order in the nuclear displacements. Furthermore, the evaluation of the off-diagonal elements of the potential matrix is based on a description of the electronic states in terms of a small active space of frontier-orbitals only. This is necessary to correctly capture the spin-structure of the states involved in fission, at the expense of the representational power of the basis used. In addition, these wave functions are constructed from Kohn-Sham orbitals which are eigenfunctions of the non-interacting Kohn-Sham system rather than the full electronic Hamiltonian. Since these orbitals are expressed in terms of Gaussian basis functions centered on the nuclei we also do not capture Pulay terms when evaluating the off-diagonal elements of W according to Supplementary equation (1). Finally, it should be noted that CT energies from TDDFT, while not severely underestimated thanks to the use of range-separation, instead come out quite significantly higher than LE. It can be suspected that the lack of diffuse functions in the basis set and the linear-response nature of the TDDFT calculations play a role here. Both hamper the ability to capture the intra-molecular relaxation of a CT configuration.

Given all these limitations, our parametrisation of the vibronic coupling Hamiltonian should be considered semi-quantitative.

Supplementary Note 2 -Transformation of vibrational environments

For the efficient application of our TTNS method we want the Hamiltonian to assume a star topology, with the exciton system at its centre and each arm representing one independent vibrational environment mapped onto a 1D-chain. In order to achieve this we need to further analyse all coupling matrices W_n. A collection of modes ω_n belong to the same independent environment i and can be mapped onto a 1D-chain if all modes couple with the same system operator $\overline{W}_i$. This means their coupling matrix elements $W_{n,(jl)}$ can be factorised as

$$W_{n,(jl)} = \overline{W}_{(jl)} \times \lambda_n, \tag{14}$$

where $\overline{W}_{(jl)}$ is the vectorisation of the system operator and λ_n is the relative coupling strength for each mode n. This definition is equivalent to requiring $\mathrm{rank}(W_{n,(jl)}) = 1$, or that it should have only one singular value/principal component. In particular, to ensure that resulting $\overline{W}$ are symmetric and stay zero where required by symmetry, in the following we restrict (jl) strictly to the upper right triangular part including the diagonal and excluding entries which are zero across all $W_{n,(jl)}$. This yields the normalisation $\|\overline{W}_{A_1}\| = 1$ for the diagonally coupled A_1 and $\|\overline{W}\| = \sqrt{2}$ for the off-diagonally coupled environments.

Clustering. For a chain mapping of arbitrary W_n we need to find the correct partition of the modes into subsets, such that each subset fulfills above condition independently. We can find this through clustering methods, e.g. k-means clustering[3], where the cluster centroids represent the approximate (mean) optimal system coupling operator $\overline{W}_i$. We modified the algorithm to adjust the centroids weighted by λ_n to reduce approximation errors for strongly coupled modes. In particular we need to find an optimal number of i clusters, such that the approximation error is minimised. An objective t-SNE projection[4] of the couplings (Supplementary Figure 3) shows that four clusters are

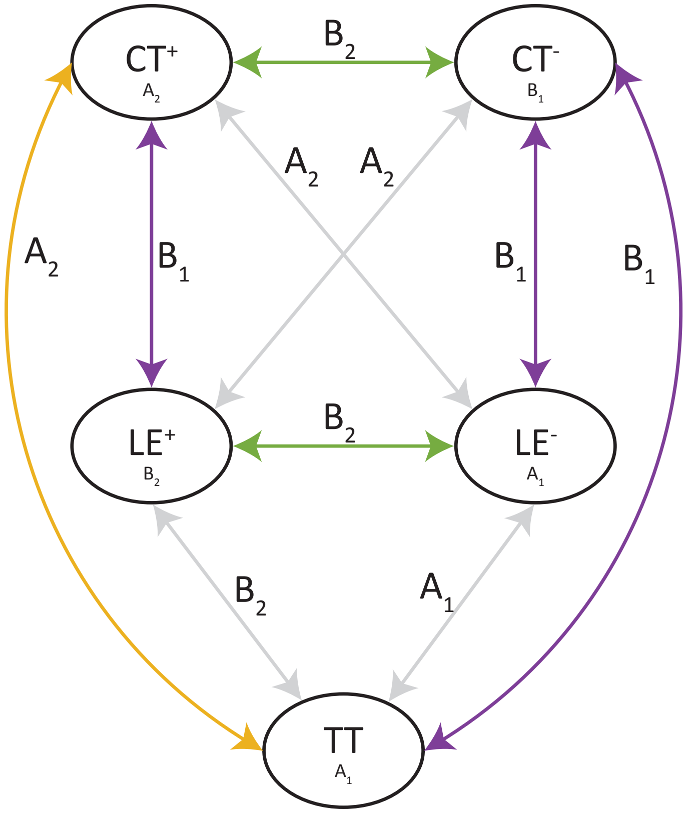

Supplementary Figure 2. The states relevant for SF and their symmetries in the point group D_{2d}. The lines indicate the vibrational coupling and the symmetry of the corresponding modes. The grey couplings involve 2 electron interactions and are vanishingly small.

the minimum, which also agree with the irreps A_1, A_2, B_1, and B_2 as obtained from group theory. However, since the clusters are fairly broad, our cluster analysis yields that at least seven clusters (coloured as in Figure 3) are necessary for an accurate representation, thus subdividing irreps A_1, B_1, and B_2.

After the clustering we can map the initial linear vibronic coupling Hamiltonian

$$\hat{H}_{LVC} = H_S + \sum_{n=1}^{318} \left[W_n \frac{(\hat{b}_n^\dagger + \hat{b}_n)}{\sqrt{2}} + \omega_n \hat{b}_n^\dagger \hat{b}_n \right], \tag{15}$$

onto the star Hamiltonian by numerically applying orthogonal polynomials or the Lanczos method[5,6] to each subset i independently. This transformation finally leads to the star Hamiltonian

$$\hat{H}_{star} = H_S + \sum_{i=1}^{7} \left[\overline{W}_i \, \|\lambda_i\| \frac{(\hat{a}_{i,0}^\dagger + \hat{a}_{i,0})}{\sqrt{2}} + \hat{H}_{c,i} \right], \tag{16}$$

$$\hat{H}_{c,i} = \sum_{k=0}^{N_i-1} \epsilon_{i,k} \hat{a}_{i,k}^\dagger \hat{a}_{i,k} + \sum_{k=0}^{N_i-2} t_{i,k} (a_{i,k}^\dagger a_{i,k+1} + hc.), \tag{17}$$

with the coupling vector $\lambda_i = (\lambda_{i_1}, \ldots, \lambda_{i_n})$, containing all couplings of assigned modes, and with the optimal coupling matrices (columns ordered as $\mathrm{TT}, \mathrm{LE}^+, \mathrm{LE}^-, \mathrm{CT}^+, \mathrm{CT}^-$):

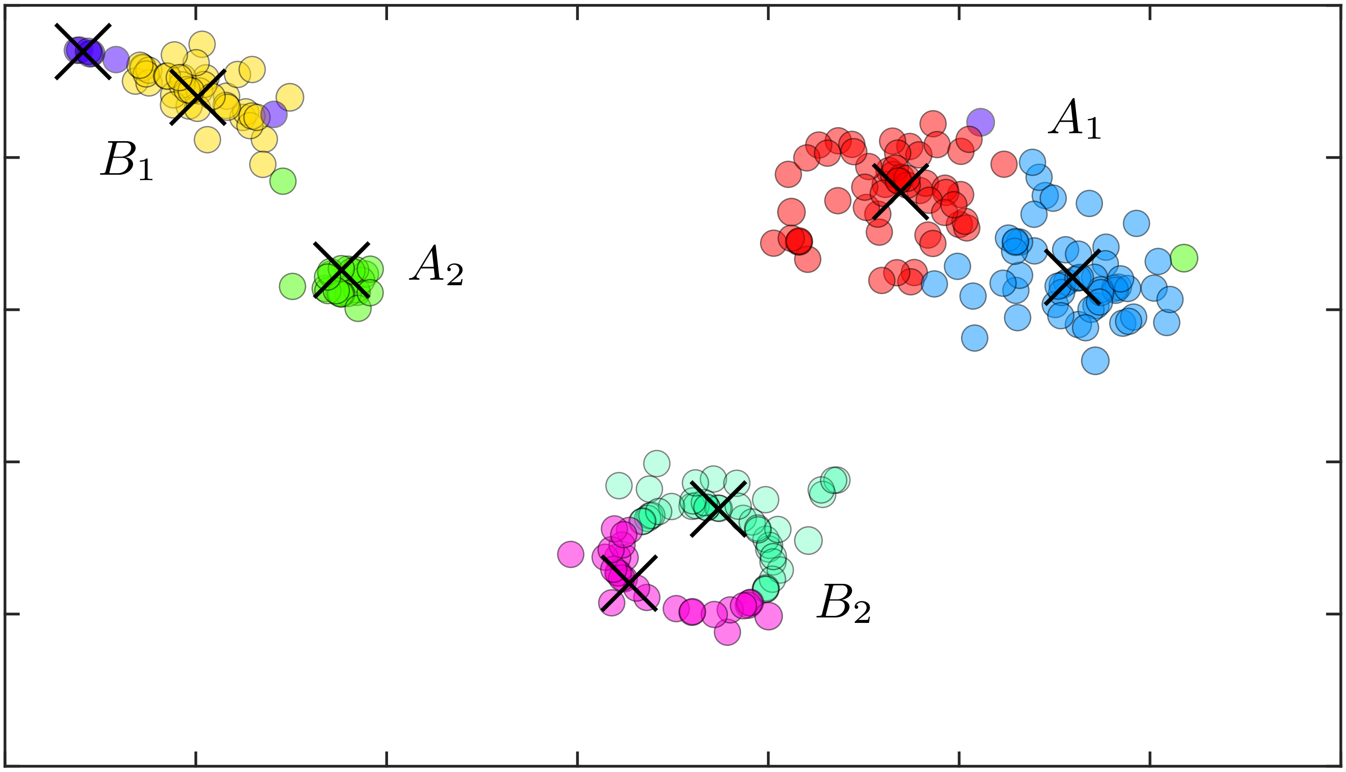

Supplementary Figure 3. 2D-Projection of the normalised vectorised coupling matrices W_n using t-SNE already reveals environments of different symmetry. The crosses represent the cluster centroids $\overline{W}_i$ and colours the assigned modes. Especially the ring structure makes it necessary to split B_2 into at least two baths.

$$\overline{W}_{A_{1,1}} = -\begin{pmatrix} 0 & & & & \\ & 0.45 & & & \\ & & 0.38 & & \\ & & & 0.57 & \\ & & & & 0.57 \end{pmatrix}, \overline{W}_{A_{1,2}} = -\begin{pmatrix} 0.75 & & & & \\ & 0.3 & & & \\ & & 0.29 & & \\ & & & 0.37 & \\ & & & & 0.37 \end{pmatrix}, \overline{W}_{A_2} = -\begin{pmatrix} 0 & & & 1 & \\ & 0 & & & \\ & & 0 & & \\ 1 & & & 0 & \\ & & & & 0 \end{pmatrix},$$
$$\overline{W}_{B_{1,1}} = -\begin{pmatrix} 0 & & & & 0.63 \\ & 0 & & -0.73 & \\ & & 0 & & 0.26 \\ & -0.73 & & 0 & \\ 0.63 & & 0.26 & & 0 \end{pmatrix}, \overline{W}_{B_{1,2}} = \begin{pmatrix} 0 & & & & 0.28 \\ & 0 & & -0.33 & \\ & & 0 & & -0.9 \\ & -0.33 & & 0 & \\ 0.28 & & -0.9 & & 0 \end{pmatrix}, \quad (18)$$
$$\overline{W}_{B_{2,1}} = -\begin{pmatrix} 0 & & & & \\ & 0 & 0.86 & & \\ & 0.86 & 0 & & \\ & & & 0 & 0.52 \\ & & & 0.52 & 0 \end{pmatrix}, \overline{W}_{B_{2,2}} = \begin{pmatrix} 0 & & & & \\ & 0 & -0.86 & & \\ & -0.86 & 0 & & \\ & & & 0 & 0.52 \\ & & & 0.52 & 0 \end{pmatrix},$$

After removing the strongly anharmonic low-frequency modes and irrelevant high frequency C-H stretches (time scales much faster than observed dynamics), 252 out of 318 modes are left. In Supplementary Table III we give all modes out of the 252 which contain at least 90% of the total coupling in each environment. In Supplementary Table IV we give the frequencies and coupling of the first sites, the reaction coordinates (RC) of each environment after chain transformation.

This star Hamiltonian model minimises the number of nearest neighbours to two for all vibrational modes and 7 for the electronic system. This is an essential optimisation needed to allow efficient TTNS simulations. Furthermore,

Supplementary Table III. All modes containing at least 90% of the total coupling

	No.	ω/cm^{-1}	λ/cm^{-1}	No.	ω/cm^{-1}	λ/cm^{-1}
$A_{1,1}$	28	129.8	398.5	40	259.5	198.1
	42	269.2	479.3	60	471.3	-116.9
	82	579.6	-712.3	88	613.7	112.6
	98	706.0	495.3	146	991.7	167.9
	178	1190.0	-175.4	200	1339.6	150.8
	225	1464.6	-270.9	228	1469.2	597.9
	236	1505.6	-326.3	260	1595.6	242.7
	266	1654.6	-282.4	271	1692.1	-338.9
$A_{1,2}$	116	805.2	1094.8	158	1036.0	-552.1
	174	1164.9	536.7	182	1219.4	537.7
	186	1232.4	-850.0	190	1265.1	974.3
	192	1271.4	1010.4	208	1372.5	985.2
	214	1426.0	1217.4	216	1445.3	-1804.3
	256	1579.0	-2285.9			
A_2	24	122.4	-206.8	38	248.0	263.0
	50	302.3	323.4	66	493.3	92.2
	100	726.3	136.2	112	783.1	-111.8
	120	873.7	-53.7	134	937.9	222.4
$B_{1,1}$	35	234.9	-148.4	39	250.6	1137.4
	61	479.2	-1157.9	65	484.9	439.0
	99	720.8	493.0	111	782.7	214.7
	133	921.5	389.9	135	939.6	305.9
$B_{1,2}$	25	122.6	410.0	49	301.7	-475.8
$B_{2,1}$	41	268.2	-373.6	83	587.8	-242.6
	85	596.7	123.1	95	696.9	230.6
	119	828.6	-131.1	159	1036.0	235.1
	173	1140.8	126.3	177	1188.2	144.1
	185	1231.1	141.8	187	1249.5	-440.6
	207	1347.7	-187.2	227	1466.9	213.2
	235	1503.2	155.8	255	1576.6	676.0
$B_{2,2}$	21	108.5	-131.4	33	217.3	-98.9
	51	324.6	94.4	81	572.2	298.5
	91	638.6	174.8	113	797.6	545.7
	181	1218.6	-146.7	191	1270.6	-162.5
	209	1384.4	202.7	215	1435.2	-600.8
	226	1464.7	-163.0	237	1522.9	78.9
	265	1654.1	100.5	272	1692.1	95.7

Supplementary Table IV. Reaction coordinate frequencies and couplings

Irrep.	ω/cm^{-1}	λ/cm^{-1}	Irrep.	ω/cm^{-1}	λ/cm^{-1}
$A_{1,1}$	903.4	1454.6	$A_{1,2}$	1377.3	4012.6
$B_{1,1}$	450.1	1851.5	$B_{1,2}$	225.4	628.2
$B_{2,1}$	1193.7	1083.9	$B_{2,2}$	1079.4	986.4
A_2	428.0	560.5			

the RCs contain valuable information about the collective action and effect of their respective environment. The RC coupling and frequency indicate total amount of coupling and weighted average frequency of all represented modes. This allows estimation of time scales and coupling regime. Additionally, expectation values of the RC's occupation and displacement represent weighted mean values for all constituent modes and can be used as a good proxy for the environment's collective state. This greatly reduces the amount of observables that have to be considered and supports the correlation with observables of the electronic system.

Supplementary Note 3 - Tree Tensor Network States

The presented simulations were performed with the time-dependent variational principle (TDVP) for matrix product states (MPS) with an optimised boson basis (OBB) as described in detail in Refs. 6–8. To accommodate the extended structure of the DP-Mes Hamiltonian H_{star}, with seven chains coupling to the electronic system, we generalised our previously described method to Tree Tensor Network States (TTNS), the higher dimensional generalisation of an MPS, which is structurally similar to the hierarchical tucker format[9], commonly used in the multi-layer multi-configurational time-dependent Hartree-Fock (ML-MCTDH) algorithm[10]. We will briefly outline the features of the algorithm, for more information we refer to Refs. 6–8, 11–13.

An MPS can approximate a many-body wave function

$$|\Psi\rangle = \sum_{\{n_k\}=1}^{d_k} \Psi_{n_1,\ldots,n_L} |n_1, \ldots, n_L\rangle, \tag{19}$$

where n_k indexes the local Hilbert space of site k with local dimension d_k, by decomposing the single rank-L tensor $\Psi_{n_1,\ldots,n_L}$, containing all probability amplitudes, into L rank-3 tensors $A_{l_k,r_k,n_k} = A^{n_k}_{l_k,r_k} \in \mathbb{C}^{D_{k-1}\times D_k \times d_k}$,

$$|\Psi_{MPS}\rangle = \sum_{\{n_k\}=1}^{d_k} A^{n_1} A^{n_2} \cdots A^{n_L} |n_1, \ldots, n_L\rangle \tag{20}$$

by means of a low-rank tensor approximation, for the case of a 1D chain Hamiltonian of length L. The bond dimensions D_k directly relate to the maximum amount of entanglement between the site k and its neighbours which can be encoded by the MPS. In order to optimally capture the entanglement arising in the time-evolution of our model, with the smallest possible D_k, we advanced our MPS tensor network to a TTNS (see Supplementary Figure 4).

Entanglement Renormalisation Tensors. In particular, we shape the TTNS such that each tensor represents one physical (local) site of H_{star}. The tensor connections follow the coupling structure in H_{star} to minimise inter-site entanglement. Since the central tensor (blue shaded in Supplementary Figure 4) connecting the system (red) with all seven environments (green) grows rapidly with increasing bond dimension (D^7), we decompose it into a tree-network of six entanglement renormalisation tensors (ER-nodes) (blue). The optimal network structure is determined by analysing the entanglement within the original core tensor in a preceding, expensive calculation. The key insight is that although this calculation of the star MPS network with $D \in [5, 8]$ is highly inaccurate, the core tensor still exhibits the major entanglement structure between the environments. By calculating the von Neumann entanglement entropy of all possible decompositions of this tensor, it is possible to find an optimal tree-network which minimises the occurring entropies (see Supplementary Figure 4).

The ER-nodes help to capture inter-environment entanglement and pass on only the most relevant effective multi-environment states. This way we can reduce the number of free parameters from D^7 down to $6D^3$ and turn the initial exponential scaling into an almost linear scaling in the number of environments. This technique is key to enable efficient simulation for > 3 environment models with high accuracy. For comparison, while in the star MPS without ER-nodes we can reasonably simulate with up to $D \in [5, 10]$ between the system and each environment, in the TTNS we can use $D_{Node} \in [50, 80]$ while still gaining a speed-up and higher accuracy (see Supplementary Figure 5).

Time-evolution with TDVP. For the time-evolution of the TTNS we decided to use the TDVP, despite previous work reporting the time-evolving block decimation (TEBD) method for TTNS[13]. TEBD involves a substantial amount of excess operations for index swaps in order to apply nearest neighbour Trotter gates which would be less feasible for the ER-tree network. Additionally TDVP offers a better complexity scaling than TEBD due to its accurate 1-site integrator, allowing larger bond dimensions. Importantly, the TTNS does not contain loops, which are typical problems for PEPS and MERA[14], it can be properly normalised and TDVP can be applied. Since we perform TDVP in a similar way as already described in[6,8,15], we will only give a brief outline here.

Although the TTNS seems to consist of functionally different components (ER-tensors, MPS chains, OBB matrices), we can identify them as nodes having one parent and multiple child tensors. Since the quasi 1D structure of the tree

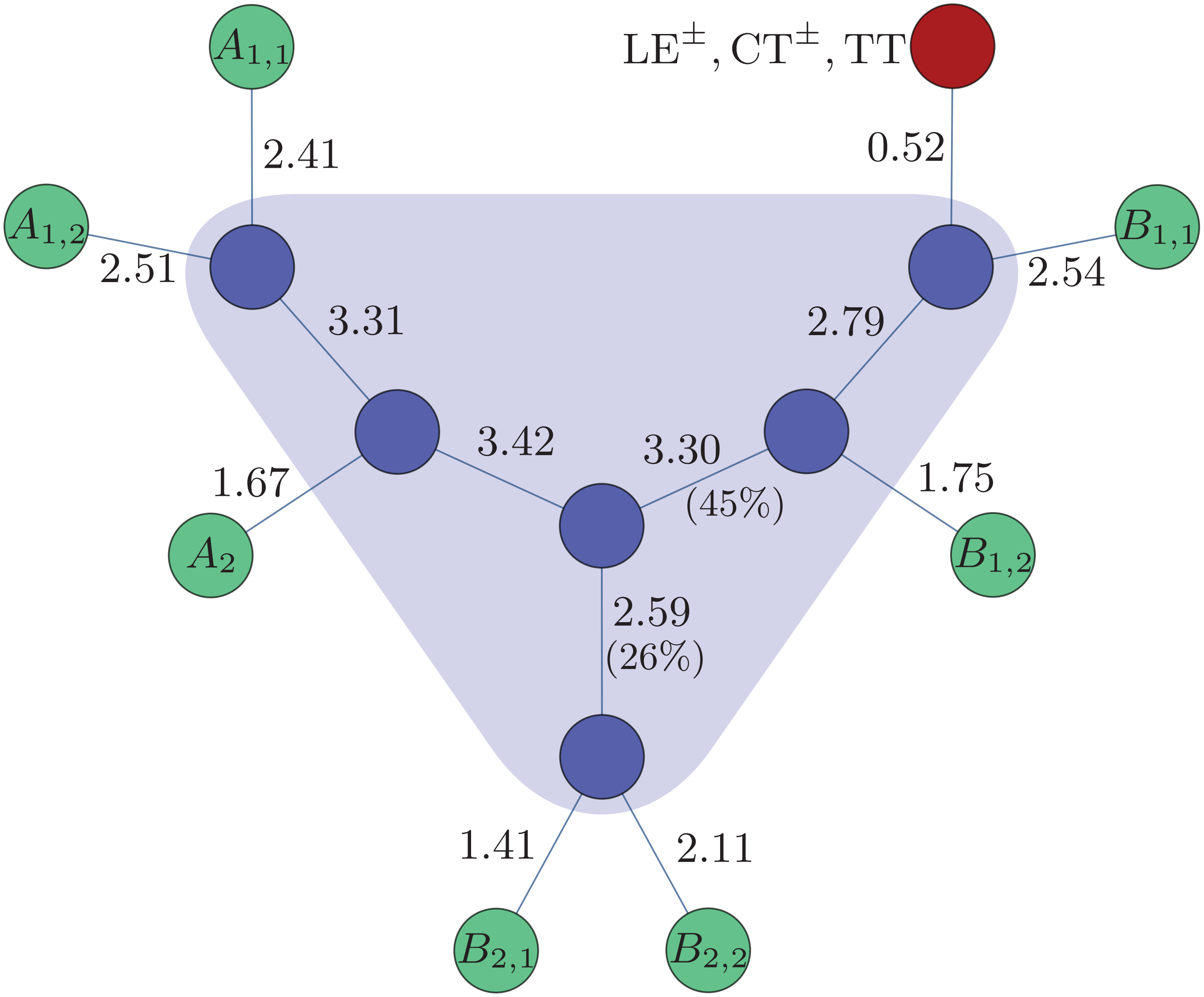

Supplementary Figure 4. The TTNS tree used to simulate the model for DP-Mes. The root node (red) represents the five-level exciton system comprising TT, $LE^{\pm}$, and $CT^{\pm}$. The seven vibrational chain environments (green) are connected to the system through a tree network of entanglement renormalising tensors (blue). The long-time entanglement entropies of each bond are indicated next to the edges, as well as two example entanglement compression rates. This tree structure optimises the inter-chain entanglement such that only a small number of relevant environmental states are coupled to the exciton system, leading to a reduced bond dimension without reducing accuracy.

ensures correct TTNS normalisation we can use the insights from Supplementary Reference 6 and generalise it to nodes with more than one child. We recursively define the time-evolution of a single node as:

1. Evolve node $t \to t + \frac{\Delta t}{2}$
2. for $i = \{1 \text{ to } N_{\text{child}}\}$
   (a) Create C to child node i and evolve $t + \frac{\Delta t}{2} \to t$
   (b) Contract C with child i and evolve $t \to t + \Delta t$
   (c) Create C from child i and evolve $t + \Delta t \to t + \frac{\Delta t}{2}$
   (d) Contract C from child with node
3. Evolve node $t + \frac{\Delta t}{2} \to t + \Delta t$

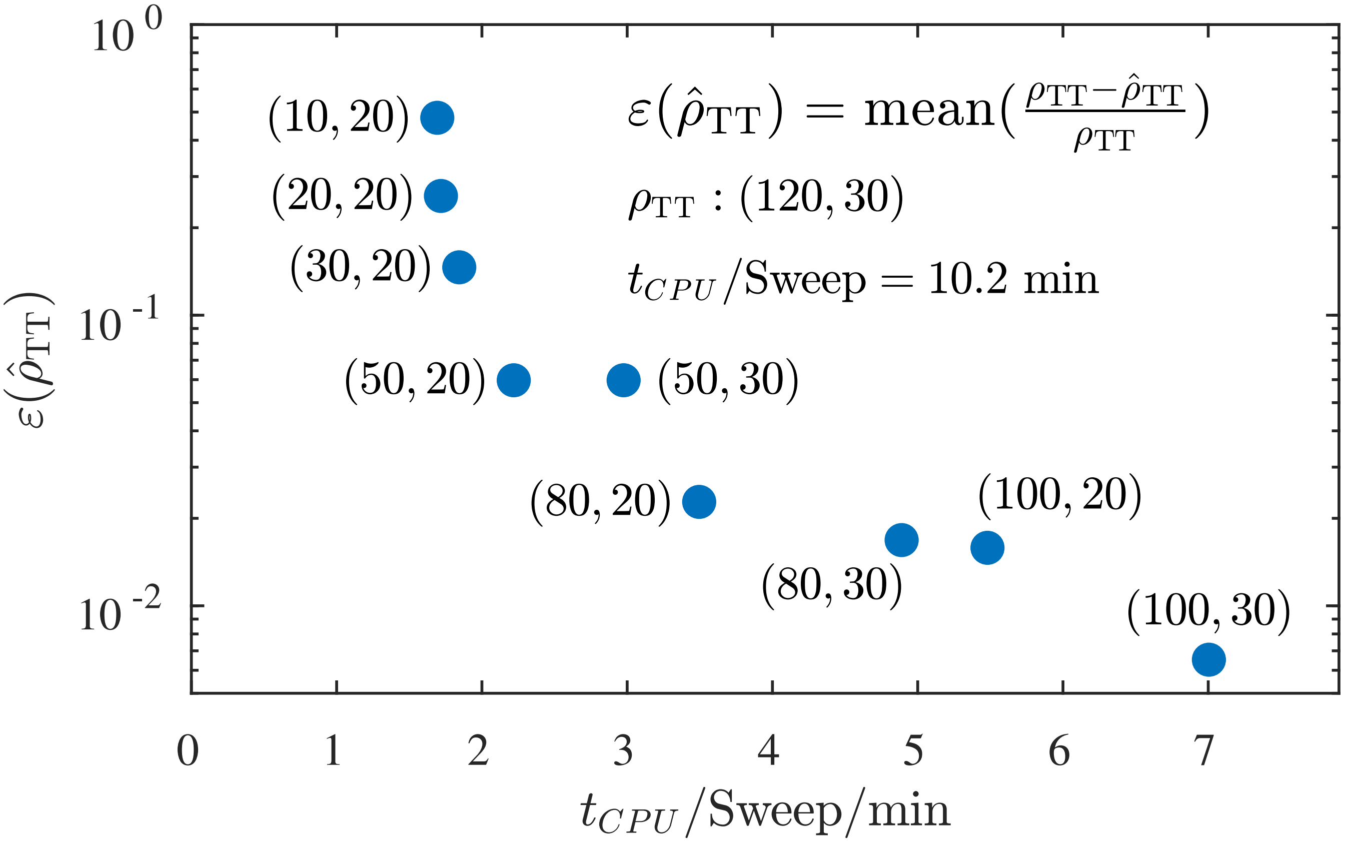

Supplementary Figure 5. Convergence in TTNS bond dimensions. Relative mean error ε of $\hat{\rho}_{\mathrm{TT}}$ with different maximum $(D_{\mathrm{Node}}, D_{\mathrm{Chain}})$ against ρ_{TT} with $(120, 30)$, plotted against CPU time. The optimal trade-off is achieved for (80,20), where $\varepsilon < 3\%$ is sufficiently small.

Where we denote C the center matrix obtained by SVD. Starting this recursive scheme from the root node with a pre-defined ordering of child nodes constitutes a sweep. To obtain a symmetric second-order integrator with error of order $\mathcal{O}(\Delta t^3)$, we sweep through the tree such that each tensor is updated twice by a time step of $\Delta t/2$.

The effective Hamiltonians needed for the time-evolution of a node are obtained in a very similar way as for the propagation of a chain tensor, by contracting the Hamiltonian with all MPS tensors which are not being propagated in the current update. This algorithm generalises and applies very naturally to ER-tensors since they can also be seen as time-dependent tensors for which we can calculate an effective Hamiltonian. To apply the matrix exponential of the effective Hamiltonians to a tensor during the update, we use a customised Krylov subspace integrator based on EXPOKIT[16].

Finally, this scheme can also be applied recursively to any tree tensor network with more layers. This allows in principle the simulation of arbitrary Hamiltonians which are representable as a tree without loops.

Supplementary Note 4 - Convergence of Simulation

We assured sufficient convergence of presented simulations by sweeping the parameters limiting the maximum bond dimensions $D_{\mathrm{Node,max}}$ and $D_{\mathrm{Chain,max}}$ and comparing the results. As measure of accuracy we compare simulations with pairs $(D_{\mathrm{Node,max}}, D_{\mathrm{Chain,max}})$ to an expensive benchmark simulation by taking the relative mean absolute error ε of the TT population kinetics. To find the performance optimum as balance between accuracy and computation time we present the error ε against the CPU time needed to propagate the TTNS to the next timestep (see Supplementary Figure 5). We chose as benchmark $(120, 30)$ since results converged sufficiently and $t_{CPU}/\mathrm{Sweep} = 10.2\,\mathrm{min}$ was the upper limit to finish a simulation within 2 weeks. We found that the accuracy is mainly limited by $D_{\mathrm{Node,max}}$ since any $D_{\mathrm{Chain,max}} \in [20, 30]$ yielded very similar errors. With $\varepsilon < 3\%$, the optimal truncation bounds were set to $(80, 30)$ which gave a total CPU time of 4 days on a single core of an Intel Xeon E5-2670 for the simulation of 1 ps.

Supplementary Note 5 - Dynamic energy surfaces

One of the central operators relevant for the time-evolution of the TTNS and for finding the ground state in DMRG is the effective Hamiltonian of a site. This operator, constructed by contracting $\hat{H}$ with all tensors except for the tensor of the chosen site, contains all information relevant for the time-evolution of the site tensor. It is this remarkable property which allows us to gain deeper insights of the dynamics and its pathways. Here we will outline how we can derive the potential energy surface (PES) and the total energy surface (TES) from the effective Hamiltonian for the exciton system, as well as the corresponding adiabatic and non-adiabatic basis states.

To introduce the concept we derive the energy surfaces of a simplified example (quantum double well). In a single mode spin-boson model (SBM)

$$\hat{H} = H_S + \lambda\sigma_z \frac{\hat{a}^\dagger + \hat{a}}{\sqrt{2}} + \omega_0 \hat{a}^\dagger \hat{a}, \tag{21}$$

with spin Hamiltonian $H_S = \frac{\Delta}{2}\sigma_x + \frac{\epsilon}{2}\sigma_z$, tunnelling rate Δ, coupling λ, and mode frequency ω_0, which is similar to the quantum Rabi model in case of vanishing energy level bias ϵ, we can obtain the operator for the potential energy $\hat{V}$ by subtracting the kinetic energy $\hat{T} = \frac{\omega_0}{2}(\hat{P}^2 - \frac{1}{2})$

$$\hat{V} = H_S + \lambda\sigma_z \hat{Q} + \frac{\omega_0}{2}\left(\hat{Q}^2 - \frac{1}{2}\right), \tag{22}$$

where we used the mass-frequency normalised coordinates $\hat{Q} = \frac{\hat{a}^\dagger + \hat{a}}{\sqrt{2}} = \hat{x}\sqrt{m\omega}$, $\hat{P} = i\frac{\hat{a}^\dagger - \hat{a}}{\sqrt{2}} = \frac{\hat{p}}{\sqrt{m\omega}}$ and divided the zero point energy equally on $\hat{T}$ and $\hat{V}$. In the next step we replace bath operators by their scalar values $\hat{Q} \to Q, \hat{P} \to P$

$$H(Q,P) = H_S + \lambda\sigma_z Q + \frac{\omega_0}{2}\left(P^2 + Q^2 - 1\right), \tag{23}$$

$$V(Q) = H_S + \lambda\sigma_z Q + \frac{\omega_0}{2}\left(Q^2 - \frac{1}{2}\right), \tag{24}$$

where the energies are now functions of the phase space coordinates (Q, P). Plotting the eigenvalues of $V(Q)$ against Q gives the adiabatic PES. The corresponding eigenvectors map the diabatic spin states $|\uparrow\rangle, |\downarrow\rangle$ into the adiabatic basis. Similarly, the eigenvalues of $H(Q,P)$ give the non-adiabatic TES and the eigenvectors form a non-adiabatic basis.

In a similar fashion we can derive the potential $\hat{V}_{star}$ from $\hat{H}_{star}$ (Supplementary equation 16) for DP-Mes by subtracting the kinetic energy terms $\hat{T}_{i,k} = \frac{\epsilon_{i,k}}{2}\left(\hat{P}_{i,k}^2 - \frac{1}{2}\right)$

$$\hat{V}_{star} = H_S + \sum_{i=1}^{7}\left(\overline{W}_i \|\lambda_i\| \hat{Q}_{i,0} + \hat{V}_{c,i}\right), \tag{25}$$

$$\hat{V}_{c,i} = \sum_{k=0}^{N_i-1} \hat{V}_{i,k} + \sum_{k=0}^{N_i-2} t_{i,k}\left(a_{i,k}^\dagger a_{i,k+1} + hc.\right), \tag{26}$$

$$\hat{V}_{i,k} = \frac{\epsilon_{i,k}}{2}\left(\hat{Q}_{i,k}^2 - \frac{1}{2}\right). \tag{27}$$

At this point, it would not be useful to replace the bath operators by scalar phase space coordinates $(\hat{Q}_{i,k}, \hat{P}_{i,k}) \to (Q_{i,k}, P_{i,k})$ since this would yield a 500-dimensional coordinate space which is impossible to analyse. Even the restriction to the RC only would still result in a 7D PES or 14D TES. Instead, we propose to only evaluate the operators along the trajectory taken by the time-evolved wave function

$$|\Psi(t)\rangle = \sum_{l=1}^{d_s}\sum_{k=1}^{D} A_{1k}^l(t)\,|l\rangle\,|\phi_k(t)\rangle, \tag{28}$$

which unravels the multi-dimensional energy surfaces into a single dimension, with time as the generalised reaction coordinate. Here, A_{1k}^l is the TTNS tensor for the electronic system and we know from the Schmidt decomposition that there are as many bath states $|\phi_k(t)\rangle$ as there are system states $|l\rangle$ for maximum system-bath entanglement.

Each $|\phi_k(t)\rangle$ can be interpreted as a particular bath configuration (displacement) or superposition of multiple bath configurations. We evaluate $\hat{H}_{star}$ and $\hat{V}_{star}$ by contracting them with the time-dependent bath-states

$$H_{ik}^{eff}(t) = \langle \phi_i(t)|\hat{H}_{star}|\phi_k(t)\rangle \,, \tag{29}$$

$$V_{ik}^{eff}(t) = \langle \phi_i(t)|\hat{V}_{star}|\phi_k(t)\rangle \,, \tag{30}$$

where $(H_{ik}^{eff})_{jl} = H_{ijkl}^{eff} = (H^{eff})_{(ij)(kl)}$ is the effective Hamiltonian of the system tensor A_{1k}^{l}. To obtain surfaces similar to the ones described in the single-mode SBM, we would need to diagonalise $H_{ii}^{eff}(t)$ and $V_{ii}^{eff}(t)$, yielding the TES and PES created by $|\phi_i(t)\rangle$ only. However, to understand the observed dynamics with strong system-environment entanglement, where the Born-Oppenheimer approximation breaks down, this picture is insufficient since there are also non-vanishing terms for $j \neq l$coupling different bath states. In order to account for them, we diagonalise the full effective operators $H^{eff}(t), V^{eff}(t) \in \mathbb{C}^{D\cdot d_s \times D\cdot d_s}$

$$H^{eff}(t) = U_H(t)E_H(t)U_H^{\dagger}(t), \tag{31}$$

$$V^{eff}(t) = U_V(t)E_V(t)U_V^{\dagger}(t), \tag{32}$$

yielding the non-adiabatic basis $U_H(t)$ and corresponding TES in $E_H(t)$, and the adiabatic basis $U_V(t)$ with PES in $E_V(t)$.

With access to the basis maps $U_H(t)$ and $U_V(t)$ we can also obtain the TES in the adiabatic basis and PES in the non-adiabatic basis as the diagonals of

$$\tilde{E}_H(t) = U_V^{\dagger}(t)H^{eff}(t)U_V(t), \tag{33}$$

$$\tilde{E}_V(t) = U_H^{\dagger}(t)V^{eff}(t)U_H(t), \tag{34}$$

which may also contain couplings since they are not purely diagonal any more.

In addition to the PES and TES landscape we want to obtain information about the population of each surface in order to track the pathway of SF. Each system wave function associated with a bath state j can be interpreted as an individual wave packet on the respective potential. By rotating the vectorised system tensor wave function $A \in \mathbb{C}^{D\cdot d_s}$ into the basis defining the PES/TES

$$\tilde{A} = U^{\dagger}A, \tag{35}$$

we can get the probability amplitude $\tilde{A}_k$ on each surface k. The population $\tilde{P}_k$ is then given by the element-wise product

$$\tilde{P}_k = \tilde{A}_k\tilde{A}_k^{*}. \tag{36}$$

Furthermore we can calculate the contribution of each diabatic system state i on each surface k by using

$$\tilde{P}_{i,k} = \sum_{j=1}^{D} U_{(ji),k}U_{(ji),k}^{*}\tilde{P}_k. \tag{37}$$

Since this expression does not include coherences between surface (adiabatic) states, it yields incorrect diabatic populations when summing over k and can only be taken as an estimate. Vice-versa, we can calculate the contribution of each surface k to each diabatic population P_i via

$$P_{k,i} = \sum_{j=1}^{D} U_{(ji),k}U_{(ji),k}^{*}P_i. \tag{38}$$

which will yield incorrect surface populations when summing over i since the coherences between diabatic states are not included.

To give an example, we present in Supplementary Figure 6 the TES (a,c) and PES (b,d) plotted with respect to two different basis states. In particular, we distinguish between the non-adiabatic energy eigenstates U_H of H^{eff} (a,b), the adiabatic states U_V (c,d), and the diabatic basis of the electronic system TT, LE$^{\pm}$, CT$^{\pm}$(colour of fills). The filled areas indicate by how much the surface is populated, while the colours give an estimate of diabatic mixing.

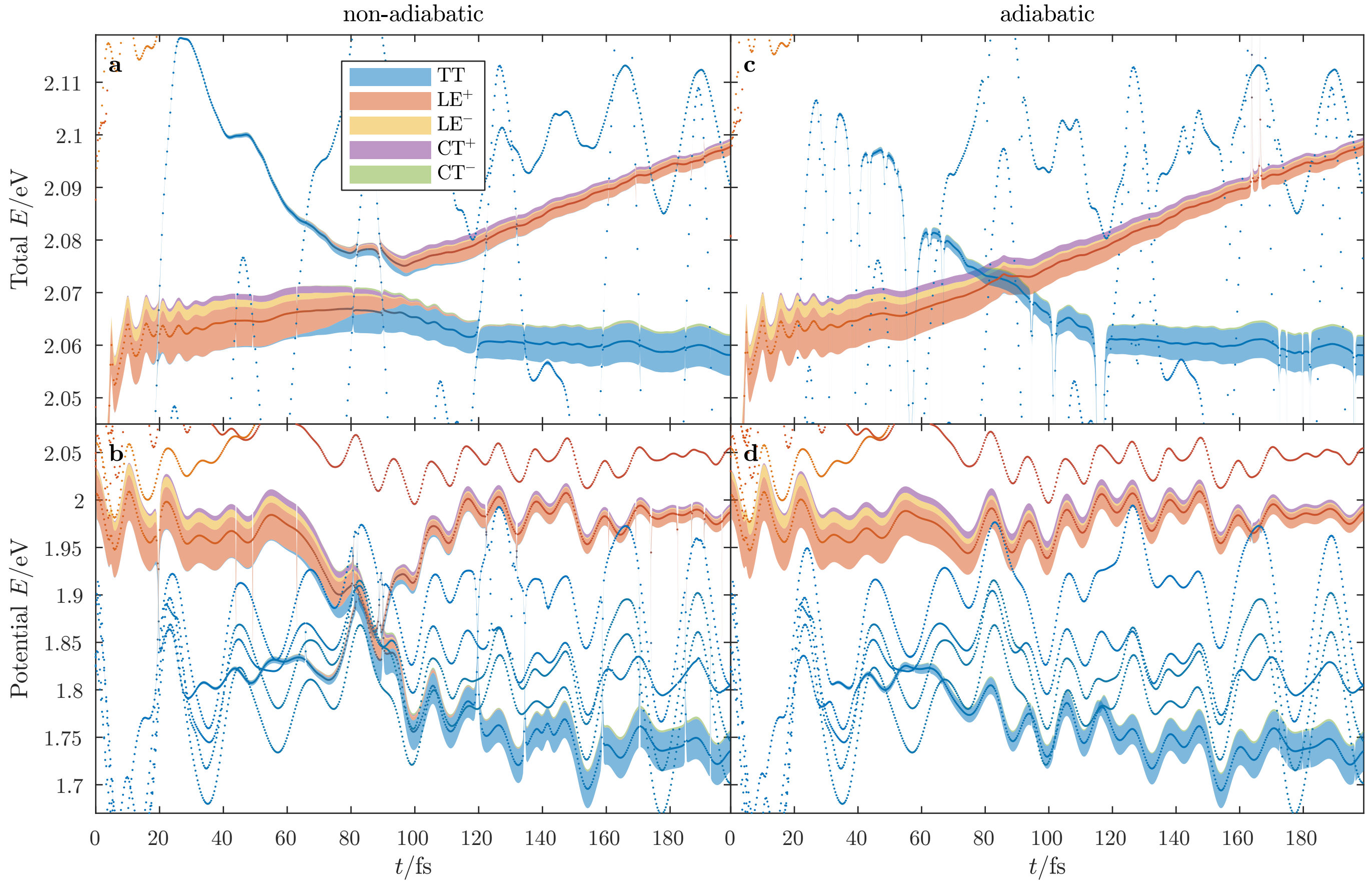

Supplementary Figure 6. Population of the TES (a) and PES (b) in the non-adiabatic basis and the TES (c) and PES (d) in the adiabatic basis within the first 200 fs. The surfaces (dots) are coloured according to the mixing of electronic states. Full population (fills) corresponds to 15% of the plot height. The amount of diabatic electronic population on each surface is highlighted with patches.

Supplementary Note 6 - Supporting energy surface results

The SF dynamics mainly occupy two relevant energy surfaces, one with major LE^+ and the other with TT character, which we will focus our discussions on. The adiabatic PES (Supplementary Figure 7d) of TT is consistently below the LE^+ PES exhibiting the absence of any conical intersection. The dynamics of each PES are only slightly downhill with a mean reorganisation of 80 meV of LE^+ and 90 meV of TT.The non-adiabatic TES (Supplementary Figure 7a) exhibits almost iso-energetic dynamics at 2.06 meV with a central avoided crossing (AC) at 80 fs. Transforming the PES into the non-adiabatic states (Supplementary Figure 7b) we see a smooth descent from the LE^+ to the TT surface indicating the exothermic SF without an activation barrier. Rotating the TES into the adiabatic basis (Supplementary Figure 7c) we can identify the actual crossing of the two coupled surfaces. In the following we detail main observations leading to SF and compare them to environmental dynamics and RC properties to investigate the mechanism.

At 20 fs the unoccupied TT TES is pushed above the LE^+ (Supplementary Figure 7a) through interaction with the B_{22} and A_{12} environments, as confirmed by comparison with their TT-projected RC displacements (Supplementary Figure 7b). While a larger $\langle x_{RC} \rangle$ in B_{22} raises the TT energy, the effect of A_{12} is opposite as evident from the bottom three TT surfaces which move anti-parallel to the displacement. We can find similar timescales in the eigenfrequencies of the respective RCs of $\omega_{RC,A_{12}} \approx 1377\,\mathrm{cm}^{-1} \sim 24$ fs and $\omega_{RC,B_2} \approx 1100\,\mathrm{cm}^{-1} \sim 30$ fs, confirming our observations. The AC at 20 fs with a gap of 6 meV is traversed rapidly by the TT, leading to almost no population exchange, while the AC at 80 fs with a gap of 12 meV seems to be the main driver of SF. The relatively weak coupling of 3 meV (half AC gap) is a result of slow B_{11} reorganisation which happens on a longer $\omega_{B_1} \approx 400\,\mathrm{cm}^{-1} \sim 83$ fs timescale leading to a larger effective vibronic coupling of 6 meV at 80 fs. Also the lowering of the TT TES is correlated with the excitation of the B_{11} RC, which leads to traversing the crossing at the same time as the effective vibronic coupling is maximised. The crossing velocity of the two surfaces is low enough to keep the majority of initial LE^+ population on

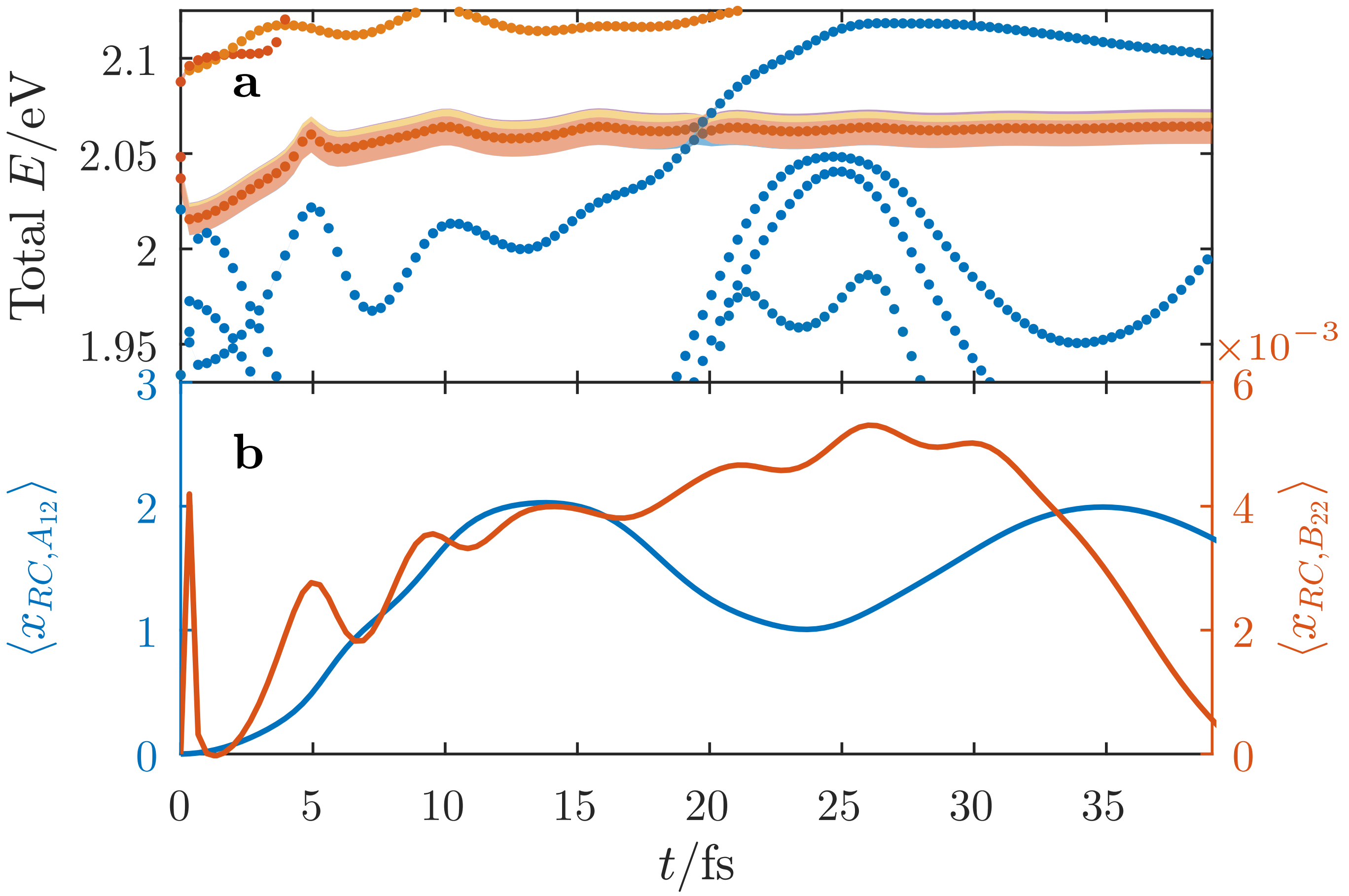

Supplementary Figure 7. The rise of the TT surface in the early time non-adiabatic TES (a) correlates with (b) the displacement of the TT-projected RC displacement of A_{12} and B_{22}.

Supplementary Table V. Reaction coordinate frequencies and couplings of the 75 mode model

Irrep.	ω/cm^{-1}	λ/cm^{-1}	Irrep.	ω/cm^{-1}	λ/cm^{-1}
$A_{1,1}$	902.1	1446.1	$A_{1,2}$	1377.5	4012.0
$B_{1,1}$	447.9	1845.1	$B_{1,2}$	225.4	628.1
$B_{2,1}$	1189.9	1073.2	$B_{2,2}$	1085.6	959.9
A_2	416.3	545.0			

the lower surfaces (see Supplementary Section).

The transfer to TT happens through change of character of the lower TES as the TT surface crosses. In the adiabatic PES it corresponds to non-adiabatic transitions between surfaces.

Remarkably, it is not (only) the Rabi-populated CT$^+$ state leading to SF since the TES TT surface does not exhibit corresponding oscillatory features. This is supported by the exclusion of the A_2 pathway in Supplementary Sec. .

Effective couplings around 6 meV have been reported previously for tetracene dimers[17], highlighting the possibility for quantitative predictive power.

Supplementary Note 7 - Dynamics with fewer modes

In order to evaluate to what extent models with less modes can give reasonably accurate results, we simulated a model based on 75 modes containing 99.7% of the coupling strength of the 252 mode model. The calculations of the truncated Hamiltonian, including only modes n with $\lambda_n > 100\,\text{cm}^{-1}$ (similar to Supplementary Table III), generates

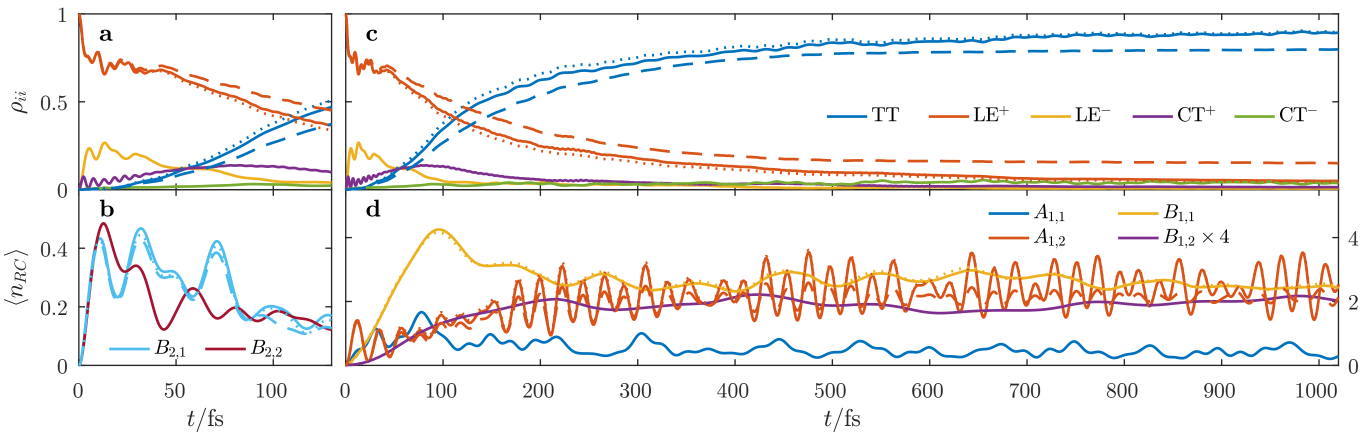

Supplementary Figure 8. Early time electronic populations (a) and environmental RC occupation dynamics (b) up to 100 fs (a) and electronic populations (c) and environmental RC occupation dynamics (d) over 1000 fs for the reduced 75 mode model of DPMES. The dynamics are very close to the full model (dotted lines) results since only weakly coupled modes were removed. Adding weak dissipation to the 75 modes via broadening yields only $\approx 80\%$ TT (dashed).

very similar dynamics (Supplementary Figure 8). This observation suggests that there is only little qualitative difference between the 252 modes and 75 modes model which is also confirmed by the parameters of the RCs in Supplementary Table V. Although this might be a reasonable argument against simulating full model dynamics, it has to be pointed out that there is only a negligible simulation speed-up with the 75 modes model. The reason is that these dynamics are causing the same amount of inter-environment entanglement that has to be compressed in the ER-network, which is the major scaling bottleneck, such that decrease in the size of each environment can be neglected.

We studied the effect of an external dissipative environment on the SF dynamics by broadening the vibrational spectra. We broaden each vibrational mode by $10\,\mathrm{cm}^{-1}$ with a Lorentzian lineshape while conserving the reorganisation energy. The resulting spectral densities are mapped onto chain environments using orthogonal polynomials as described in Supplementary Reference 6. We truncate each semi-infinite chain to a length of $L = 70$, giving a total of 490 modes, such that the exciton dynamics are unaffected by reflections from the end of the chains. The resulting dynamics show a lower TT yield of 80% along with a three times larger LE^+ population of 15%. The absence of further kinetic energy seems to limit SF to this upper value. However, as soon as the reflection from the end of the chains impact on the system past 1 ps, the TT increases again by a few percent. This suggests, that even after an equilibrium is reached, excitation of the environment, such as heat or a push pulse, could increase the yield of fission.

The super-exchange character of $\mathrm{LE}^- \xrightarrow{B_1} \mathrm{CT}^- \xrightarrow{B_1} \mathrm{TT}$ is confirmed by the appearance of $\mathrm{Im}(\rho_{\mathrm{TT,LE}^-})$ and the large static coherence $\mathrm{Re}(\rho_{\mathrm{TT,LE}^-})$ (Supplementary Figure 9). The latter manifests as a coherent superposition of TT, LE^-, and CT^- in the long-time state (> 300 fs). The mixture of this strong vibronic (dressed) state is modulated by B_1 and A_1 (Supplementary Figure 8c). All other coherences are small compared to $\rho_{\mathrm{TT,LE}^-}$, as expected for linear vibronic coupling.

The von Neumann entropy S of the RDM increases until 90 fs which shows the strong non-adiabatic character of the SF dynamics (see Supplementary Figure 9). At this point the electronic state is maximally distributed among $\mathrm{LE}^\pm$, $\mathrm{CT}^\pm$, and TT. As the TT population increases subsequently, S decreases again due to purification of the state and demixing with the environment.

Supplementary Note 8 - Rabi Dynamics

The < 20 fs dynamics are dominated by very fast vacuum Rabi-like oscillations. However, correlating system and environment observables, such as $\langle n_{RC}\rangle$ (see Figure 3) to visualise these is indicative, since expectation values are averages over microscopic states of competing dynamics, such as environmental reorganisation. By taking $\langle n\rangle$ only with respect to one of the five effective (microscopic) bath states $\langle\phi_j|\hat{n}|\phi_j\rangle$ we can single out one particular type of process. The system tensor wave function $A \in \mathbb{C}^{D\times d_s}$ tells which pairs of system and bath states are dominant.

The Rabi dynamics $\mathrm{LE}^+ \xleftrightarrow{B_1} \mathrm{CT}^+$ are happening between $|\mathrm{LE}^+, \phi_1\rangle$ and $|\mathrm{CT}^+, \phi_3\rangle$, as evident from the anti-parallel dynamics of $\rho(\mathrm{CT}^+)$ and $\langle\phi_3|\hat{n}_{B_1,RC}|\phi_3\rangle$ (Supplementary Figure 10b). We can see that $B_{1,2}$ only participates to 2% while $B_{1,1}$ has a contribution of 98%. Since the driving is very off-resonant and comparably weak, with a

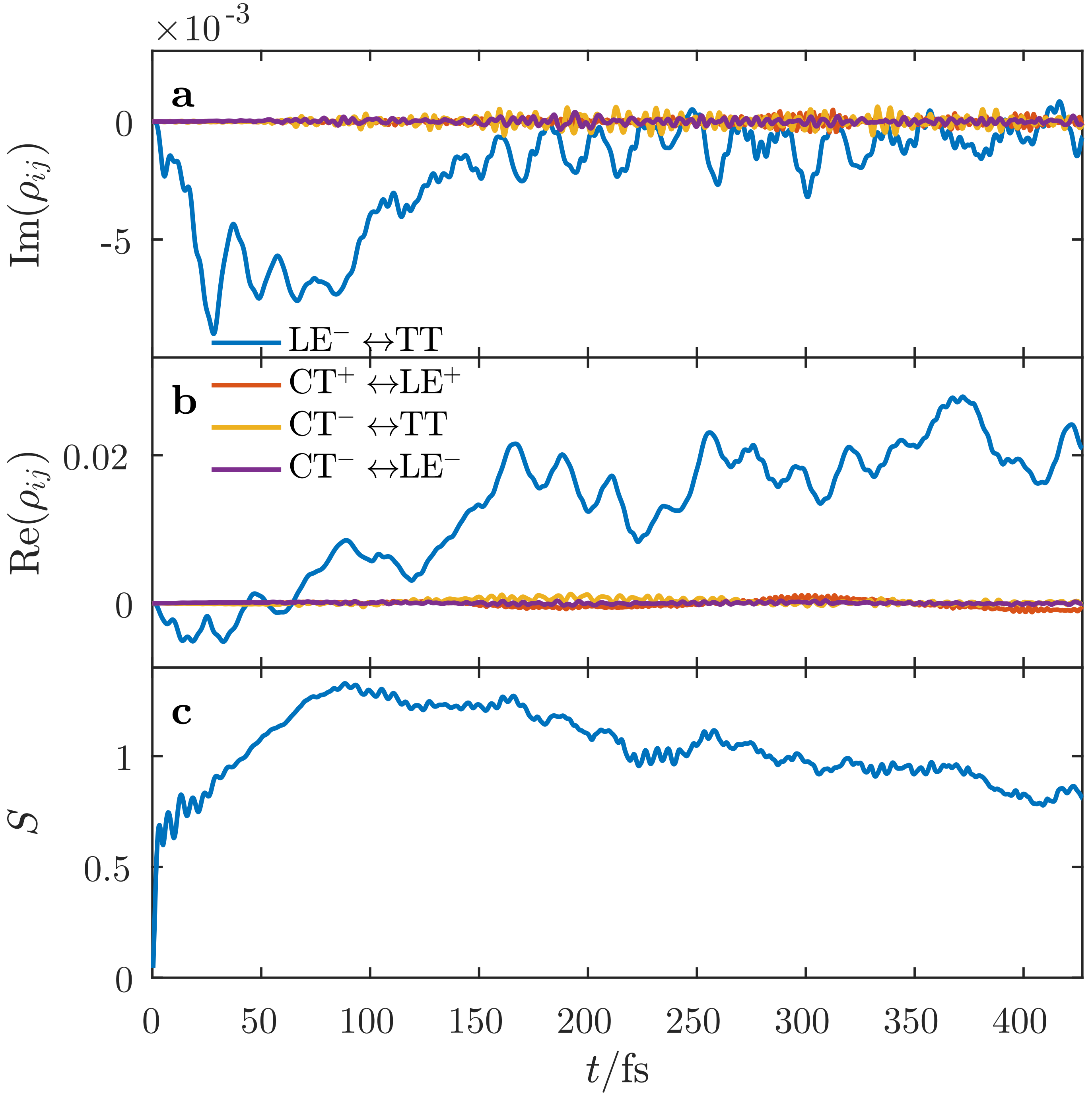

Supplementary Figure 9. Coherences and entropy of the 75 mode model. Amongst the dynamic coherences $\mathrm{Im}(\rho_{ij})$ (a) and the static coherences $\mathrm{Re}(\rho_{ij})$ (b), $\rho_{\mathrm{TT,LE^-}}$ is the by far the largest off diagonal element of the reduced density matrix, due to the super-exchange via B_1. The system von Neumann entropy S shown in (c) increases initially due to strong state mixing and environmental entanglement but subsequently decreases indicating a purification of the overall state.

RC frequency $\omega = 450\,\mathrm{cm}^{-1}$, an $\mathrm{LE}^+ - \mathrm{CT}^+$ energy gap $\Delta \approx 5500\,\mathrm{cm}^{-1} - 6500\,\mathrm{cm}^{-1}$, and coupling $\lambda \approx 1350\,\mathrm{cm}^{-1}$, the Rabi frequency Ω_R is very large ($T \approx 5.1\,\mathrm{fs} \rightarrow \Omega_R \approx 6500\,\mathrm{cm}^{-1}$). It should be noted that in this regime the counter-rotating terms cannot be neglected such that the usual rotating-wave approximation (RWA) is invalid. Thus we obtain the Rabi frequency as[18]

$$\Omega_R = \Delta\left(1 + \frac{\lambda^2}{\Delta^2}\right). \qquad (39)$$

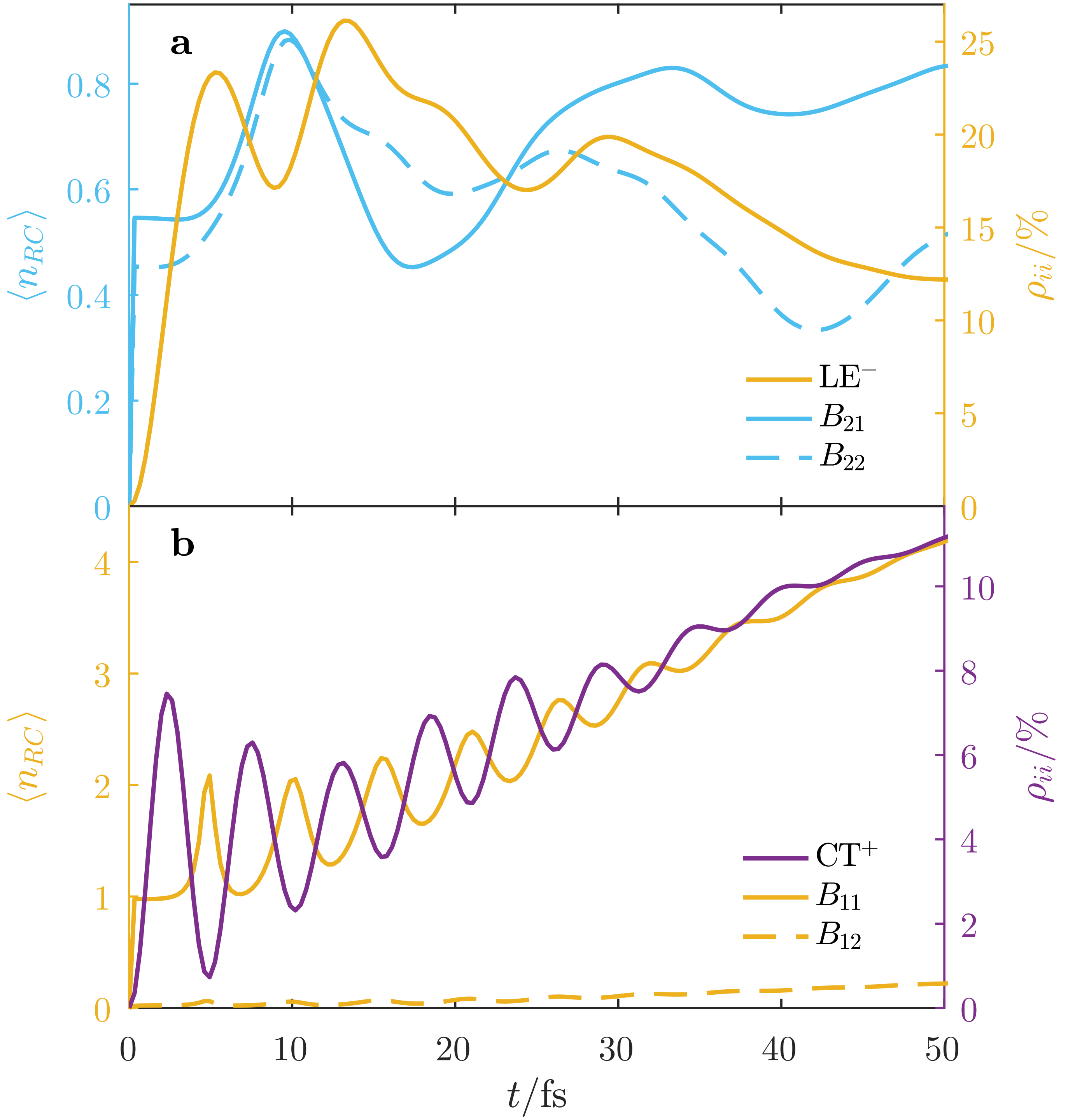

Supplementary Figure 10. Initial time Rabi-like dynamics. (a) The $\mathrm{LE}^+ \xleftrightarrow{B_2} \mathrm{LE}^-$ Rabi oscillations are equally strong in B_{21} and B_{22} and vanish already after 15 fs but create ≈ 20%LE^-. (b) $\mathrm{LE}^+ \xleftrightarrow{B_1} \mathrm{CT}^+$ undergoes Rabi dynamics mainly with B_{11}, lasting for 40 fs. The low yield of 8% indicates strong detuning.

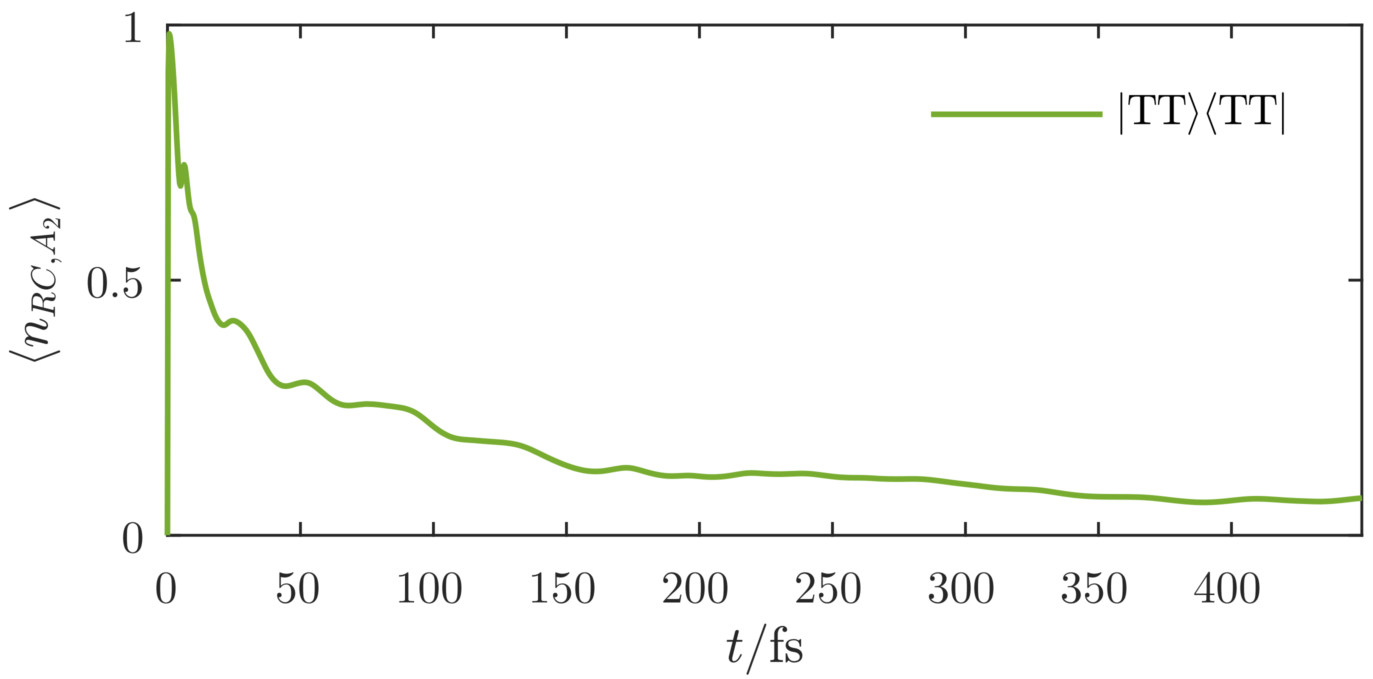

Supplementary Figure 11. A_2 RC occupation projected onto TT. Most of the initial $< 10\,\mathrm{fs}$ TT was generated via pathway I as indicated by the large $\langle n_{RC,A_2}\rangle > 0.7$. At the equilibrium state $\langle \hat{n}_{RC,A_2}\rangle \approx 0.07$ shows that only 7% of the total TT came from path I, while the rest has taken path II.

The maximum population on CT^+ can only reach 8% as confirmed by

$$P(\mathrm{CT}^+) = \frac{\lambda^2}{\Omega_R^2}. \tag{40}$$

The process $\mathrm{LE}^+ \xleftrightarrow{B_2} \mathrm{LE}^-$ involving $|\mathrm{LE}^+,\phi_1\rangle$ and $|\mathrm{LE}^-,\phi_2\rangle$ is strongly damped and lasts only for 2 periods, while rapidly generating $\approx$ 20% of LE^- (Supplementary Figure 10a). Here the two split environments are equally participating with 55% ($B_{2,1}$) and 45% ($B_{2,2}$), as seen by the initial value of the occupation.Since the detuning is much lower ($\omega \approx 1100\,\mathrm{cm}^{-1}, \Delta \approx 2314\,\mathrm{cm}^{-1} - 3950\,\mathrm{cm}^{-1}, \lambda \approx 860\,\mathrm{cm}^{-1}$) the expected Rabi frequency is lower ($\Omega_R \approx 2633\,\mathrm{cm}^{-1} - 4137\,\mathrm{cm}^{-1}$) . The maximum expected LE^- population ranges within 11% – 4% per environment, which agrees with the actually achieved 23%.

Supplementary Note 9 - Exclusion of A_2 pathway

We can confidently prove that pathway I coupled via the A_2 environment is not contributing substantially to the observed SF dynamics by looking at the TT projected occupation

$$\langle \hat{n}_{RC,A_2,\mathrm{TT}}\rangle = \langle\Psi|\, P_{\mathrm{TT}}\hat{n}_{RC,A_2}\,|\Psi\rangle\,, \tag{41}$$

where $P_{\mathrm{TT}} = |\mathrm{TT}\rangle\langle\mathrm{TT}|$ is the projector onto TT. Since the vibrationally mediated transport $\mathrm{CT}^+ \to \mathrm{TT}$ can only happen through the excitation of A_2 as $|\mathrm{CT}^+\rangle\,|0\rangle_{A_2} \to |\mathrm{TT}\rangle\,|1\rangle_{A_2}$, while pathway II via $\mathrm{LE}^- \to \mathrm{CT}^- \to \mathrm{TT}$ happens at $|0\rangle_{A_2}$, the magnitude of this TT projected occupation gives direct evidence of how much TT has come via pathway I. Initially, $\langle \hat{n}_{RC,A_2,\mathrm{TT}}\rangle \approx 1$ indicates that almost all TT was generated through path I. Subsequently this occupation drops rapidly, indicating a larger population of $|\mathrm{TT}\rangle\,|0\rangle_{A_2}$ which can only happen via path II. The final value $\langle \hat{n}_{RC,A_2}\rangle \approx 0.07$ shows that 93% of TT has been generated through path II.

Supplementary Note 10 - $\mathrm{LE}^+ \to \mathrm{TT}$ as Landau-Zener transition

We can interpret the passage through the avoided crossing in terms of a Landau-Zener passage which allows the adiabatic transition $\mathrm{LE}^+ \to \mathrm{TT}$ if the decrease in energy gap is slow enough. Based on the Landau-Zener formula for

the probability of diabatic transition

$$P_D = \exp\left(-2\pi \frac{a^2}{\hbar|\alpha|}\right), \tag{42}$$

with $a = 6\,\text{meV}$ being the effective coupling between the surfaces and $\alpha = 0.727\,\text{meV/fs}$ the rate of change of the energy gap, we can calculate a 62% transition probability which is not properly matching the observed kinetics. The formula would predict a coupling of 10 meV to yield the expected 25% transition probability. Given that the Landau-Zener formula is based on multiple assumptions which are all violated in these real dynamics, such as a time-independent diabatic coupling, linearly varying energy gap, and the perturbation parameter of the Hamiltonian being a linear function of time, it is ensuring that the found and expected effective coupling are at a comparable order of magnitude.

---

[1] Worth, G. A. & Cederbaum, L. S. BEYOND BORN-OPPENHEIMER: Molecular Dynamics Through a Conical Intersection. *Annual Review of Physical Chemistry* **55**, 127–158 (2004).

[2] Smith, M. B. & Michl, J. Singlet Fission. *Chemical Reviews* **110**, 6891–6936 (2010).

[3] Arthur, D. & Vassilvitskii, S. K-Means++: the Advantages of Careful Seeding. In *Proceedings of the Eighteenth Annual ACM-SIAM Symposium on Discrete Algorithms*, SODA '07, 1027–1025 (Society for Industrial and Applied Mathematics, Philadelphia, PA, USA, 2007).

[4] van der Maaten, L. & Hinton, G. Visualizing data using t-SNE. *Journal of Machine Learning Research* **9**, 2579–2605 (2008).

[5] Chin, A. W., Rivas, Á., Huelga, S. F. & Plenio, M. B. Exact mapping between system-reservoir quantum models and semi-infinite discrete chains using orthogonal polynomials. *Journal of Mathematical Physics* **51**, 092109 (2010).

[6] Schröder, F. A. Y. N. & Chin, A. W. Simulating open quantum dynamics with time-dependent variational matrix product states: Towards microscopic correlation of environment dynamics and reduced system evolution. *Physical Review B* **93**, 075105 (2016).

[7] Haegeman, J., Lubich, C., Oseledets, I., Vandereycken, B. & Verstraete, F. Unifying time evolution and optimization with matrix product states. *Physical Review B* **94**, 165116 (2016).

[8] Lubich, C., Oseledets, I. V. & Vandereycken, B. Time Integration of Tensor Trains. *SIAM Journal on Numerical Analysis* **53**, 917–941 (2015).

[9] Grasedyck, L., Kressner, D. & Tobler, C. A literature survey of low-rank tensor approximation techniques. *GAMM-Mitteilungen* **36**, 53–78 (2013).

[10] Wang, H. & Thoss, M. Multilayer formulation of the multiconfiguration time-dependent Hartree theory. *The Journal of Chemical Physics* **119**, 1289–1299 (2003).

[11] Schollwöck, U. The density-matrix renormalization group in the age of matrix product states. *Annals of Physics* **326**, 96–192 (2011).

[12] Szalay, S. *et al.* Tensor product methods and entanglement optimization for ab initio quantum chemistry. *International Journal of Quantum Chemistry* **115**, 1342–1391 (2015).

[13] Shi, Y.-Y., Duan, L.-M. & Vidal, G. Classical simulation of quantum many-body systems with a tree tensor network. *Physical Review A* **74**, 022320 (2006).

[14] Orús, R. A practical introduction to tensor networks: Matrix product states and projected entangled pair states. *Annals of Physics* **349**, 117–158 (2014).

[15] Lubich, C. Time Integration in the Multiconfiguration Time-Dependent Hartree Method of Molecular Quantum Dynamics. *Applied Mathematics Research eXpress* **2015**, 311–328 (2015).

[16] Sidje, R. B. Expokit: a software package for computing matrix exponentials. *ACM Transactions on Mathematical Software* **24**, 130–156 (1998).

[17] Alguire, E. C., Subotnik, J. E. & Damrauer, N. H. Exploring Non-Condon Effects in a Covalent Tetracene Dimer: How Important Are Vibrations in Determining the Electronic Coupling for Singlet Fission? *The Journal of Physical Chemistry A* **119**, 299–311 (2015).

[18] Allen, L. & Eberly, J. H. *Optical Resonance and Two-level Atoms.* Dover books on physics and chemistry (Dover, 1975).
